# The effectiveness of gluten-free dietary interventions: A systematic review

**DOI:** 10.3389/fpsyg.2023.1107022

**Published:** 2023-03-22

**Authors:** Michaela Poslt Königová, Martina Sebalo Vňuková, Petra Řehořková, Martin Anders, Radek Ptáček

**Affiliations:** Department of Psychiatry, First Faculty of Medicine, Charles University and General University Hospital, Prague, Czechia

**Keywords:** celiac disease, gluten-free diet, intervention, health-related quality of life, diet adherence

## Abstract

Celiac disease is a chronic autoimmune gastroenterological disorder in which the digestion of gluten leads to damage and constant inflammation in the small intestine. Moreover, there are associated physical and mental health problems related to celiac disease, i.e., a lower health-related quality of life and increased depression and anxiety symptoms. The only effective treatment for celiac disease is lifelong adherence to a gluten-free diet. However, researchers suggest that strict adherence to a gluten-free diet ranges from 42 to 80%, depending on the definition and method of assessment that was utilized. This review examines interventions designed for those who need to adhere to life-long dietary measures and their success in terms of increasing gluten-free dietary adherence and improving their health-related quality of life. In April 2022, the Scopus, Web of Science, PubMed and ProQuest databases were searched using the following terms: “coeliac disease” OR “celiac disease” AND “gluten free diet” AND “intervention” AND “health related quality of life” AND “diabetes.” Eight studies were suitable for this review. The studies were used to analyze different intervention techniques and their impact on gluten-free dietary adherence, quality of life, and the reasons for dietary nonadherence. The studies revealed statistically significant improvements in the knowledge base regarding celiac disease and the gluten-free diet, dietary adherence and quality-of-life satisfaction immediately after the intervention and at a three-month follow-up. Some studies were also focused on behavioral and cognitive aspects of nonadherence to dietary measures.

## Introduction

Celiac disease is a chronic, autoimmune, genetically predisposed gastroenterological disorder in which sensitivity to gluten leads to damage and constant inflammation of the small intestine (Ford et al., 2012). The digestion of gluten, the primary storage protein of wheat and other grains such as barley, rye and oats, leads to the destruction of the villi in the small intestine (Ciclitira et al., 2005) and an inflammatory response in the intestine that is detectable by blood testing (Woodward, 2011; Beaudoin and Zimbardo, 2012). Therefore, the surface of the small intestine weakens, and the ability to digest and absorb nutrients decreases significantly (Woodward, 2011). The disease is also associated with osteoporosis, fertility issues in women, diabetes, increased risk of gastrointestinal cancer and non-Hodgkin’s lymphoma (Ford et al., 2012). The global prevalence rate of celiac disease is approximately 1–1.4% (Mustalahti et al., 2010; Gujral et al., 2012; Singh et al., 2018), although the prevalence rate in Ireland and Scandinavian countries is higher, with a suggested prevalence rate of 2–2.4% (Mustalahti et al., 2010). However, because of different manifestations of the disease and the absence of screening, the exact numbers are not known but are expected to be significantly higher (Woodward, 2011; Ford et al., 2012; Green et al., 2015). Celiac disease is usually diagnosed through a blood sample and small intestine biopsy to detect villus atrophy (Ford et al., 2012).

The only effective treatment for celiac disease is lifelong adherence to a gluten-free diet. A gluten-free diet requires avoidance of all foods that contain wheat, rye, barley, and oats. There are still controversies around consumption of oats, although there are studies suggesting that the protein contained in oats is well tolerated by some of the patients with celiac disease up to a daily limit of 50-70 g of oats for adults and 20-25 g for children (Pulido et al., 2009; Comino et al., 2015; Spector Cohen et al., 2019). Strict adherence to the diet allows the intestinal and immune systems to heal. However, the response to this therapy is not satisfactory, and in approximately 20% of individuals, the symptoms prevail even after diagnosis due to nonadherence to a gluten-free diet (Ciclitira et al., 2005). There are various motives that are cited as reasons why patients with celiac disease do not adhere to the gluten-free diet as strictly as they should, and among the most frequent ones are stress associated with the diet, adverse effects of the diet on their social life, negative effects of the diet on their mood and increased anxiety due to having to maintain a gluten-free diet (Leffler et al., 2007). Moreover, the participants also stress the costs associated with a gluten-free diet, the difficulty of finding food outside of the home, and poor food quality (Leffler et al., 2007) as reasons for nonadherence. Furthermore, accidental exposure to gluten because of poor labeling or lack of information about celiac disease and the gluten-free diet by restaurant and bar staff members is also a source of increased frustration and even social anxiety in patients with celiac disease (Addolorato et al., 2008; Zysk et al., 2018).

The literature on celiac disease and its impact on physical and psychological well-being is broad. Studies that were focused on psychological problems revealed that patients with celiac disease suffer from depression, a lower health-related quality of life, and poorer overall psychological well-being and anxiety than those in the general population (Ford et al., 2012). Besides the three most common possible biological explanations why celiac disease might trigger or mask psychological problems, i.e., the malabsorption of nutrients essential for normal functioning of the brain, the immunological reaction and release of antibodies affecting the hypothalamus-pituitary–adrenal axis, and the extra-intestinal inflammation of the body (Beaudoin and Zimbardo, 2012), there are various psychological and cognitive reasons for higher incidents of comorbid psychological problems and reports of a lower quality of life due to chronic disease (Ford et al., 2012). For example, in a study by Addolorato et al. (2008), the researchers suggest that social phobias and avoidance of social situations are fairly standard, as the gluten-free diet can be frustrating and isolating, and diet restrictions might lead to difficulties in daily social relationships for many reasons (Addolorato et al., 2004, 2008). Furthermore, European studies suggest that lower adherence to a gluten-free diet and poor adaptation to the disease are correlated with anxiety and depression (Ford et al., 2012). A systematic study found that strict adherence to a gluten-free diet ranges from 42 to 80%, depending on the definition and assessment method (Dowd et al., 2014), which is far below the expected rates. In a study presented by the Finland University research center, the authors mention that the decentralization of celiac disease diagnostics, follow-up from tertiary centers to primary care and insufficient information about the disease and the diet are the reasons for those high numbers (Kurppa et al., 2013).

As mentioned above, the research was focused on the psychological burden associated with celiac disease, and a gluten-free diet is very burdensome. Because gluten-free diet adherence is essential in the improvement of physical and mental health, the goal of this review is to map intervention strategies that aim to change and improve psychological well-being for patients with celiac disease. The present review examines these interventions and their success in terms of gluten-free adherence and changes in health-related quality of life.

## Methods

The aim of the study is to give a clear picture of the interventions for patients with celiac disease to increase gluten-free diet adherence and to analyze the success of these interventions in terms of gluten-free diet adherence and changes in the health-related quality of life. This paper includes a systematic literature review that is characterized by a systematic search process to locate all relevant published work that addresses the research question as well as systematic presentation and synthesis of the findings of the results of that research (Siddaway et al., 2019). For this reason, the following databases were searched in April 2022: Scopus, Web of Science, PubMed, and ProQuest. Academic papers were selected using the following string search: Abstract or title = “coeliac disease” OR “celiac disease” AND “gluten free diet” AND “intervention” AND “health related quality of life” AND “diabetes.” The studies must have been published from 2015 onward in the English language and in full text.

Table 1: Results generated through our initial database search.

**Table 1:** Results generated through the database search.

Search term	Scopus	Web of science	PubMed	ProQuest	All
Coeliac disease	11,751	8,541	7,594	10,416	38,302
celiac disease	11,751	1708	7,594	10,416	31,469
Gluten free diet	4,332	3,205	2,755	5,339	15,631
Intervention	791,936	750,883	3,785,013	564,740	5,892,572
Health related quality of life	60,768	60,961	244,610	393,775	760,114
Diabetes	388,991	305,212	342,724	305,084	1,342,011
1 OR 2 AND 3 AND 4 AND 5 NOT 6	11	8	0	8	27
Removed duplicates	11	8	0	8	22

Records were then screened using the following exclusion and inclusion criteria.

Inclusion criteria were as follows:

1. Studies written in English language;
2. Studies published from 2015 onward;
3. Studies with male and female populations above the age of 16;
4. Studies that were focused on nonmedical interventions for celiac disease; and
5. Studies with designs that included quantitative methods or studies that reported on findings that were derived from quantitative methods.

Exclusion criteria:

1. Studies that did not provide full-text access or that could not be retrieved through our university libraries;
2. Studies that were focused only on medical interventions for celiac disease or biological explanations of associated problems (e.g., gut transplant);
3. Studies that used qualitative methods or secondary research; and
4. Studies that were review articles.

**Figure 1:**
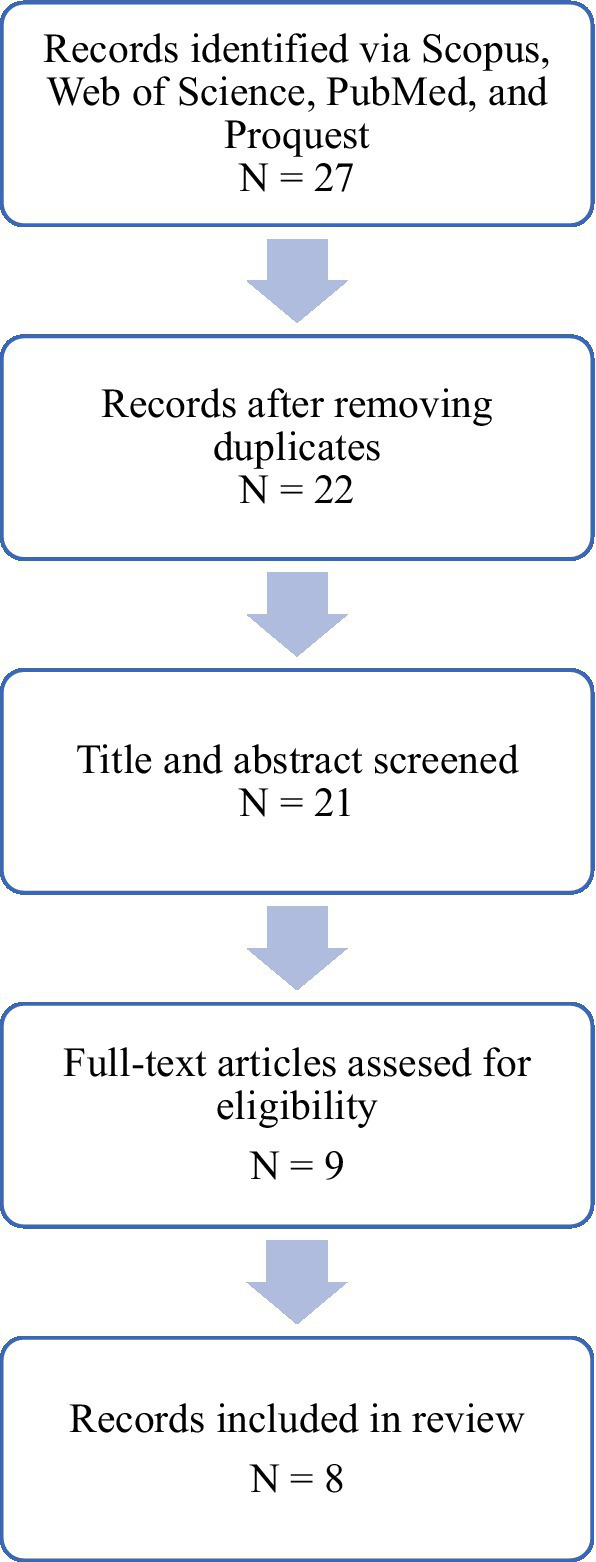
Representation of the inclusion and exclusion process.

The remaining records were included in this review (Figure 1).

In the selected databases, 27 articles were identified, out of which 6 were removed automatically because of 5 duplicates and 1 article written in Hungarian. Next, we screened the articles in terms of the type of article, and another 7 articles were removed. We then assessed the articles for their eligibility given the criteria stated above, which resulted in the inclusion of 8 studies in the review.

We evaluated the quality of articles with the modified version of the Effective Public Health Practice Project (EPHPP) quality assessment tool for quantitative studies. Questions about criteria unrelated to this review were excluded. Detailed component ranking can be found in Appendix 1.

## Results

### Results of included studies in terms of focus and demographics

Tables 2–5: The studies included in the review.

**Table 2:** Summary of the included studies.

Author	Name	Country	Study design
Akbari Namvar et al. (2022)	The effect of group-based education on gastrointestinal symptoms and quality of life in patients with celiac disease	Iran	Randomized controlled clinical trial
Dowd et al. (2022)	Effects of a 12-week HIIT + group mediated cognitive behavioral intervention on quality of life among inactive adults with coeliac disease: Findings from the pilot MOVE-C study	Canada	Randomized controlled clinical trial
Martínez-Rodríguez et al. (2021)	Effects of 12 weeks of strength training and a gluten-free diet on quality of life, body composition and strength in women with celiac disease	Spain	Randomized controlled clinical trial
Muhammad et al. (2021)	Telephone clinic improves gluten-free dietary adherence in adults with coeliac disease: Sustained at 6 months	UK	Randomized controlled clinical trial
Wolf et al. (2020)	A cooking-based intervention promotes gluten-free diet adherence and quality of life for adults with celiac disease	USA	Cross-sectional
Haas et al. (2017)	Text message intervention (TEACH) improves quality of life and patient activation in celiac disease: A randomized clinical trial	USA	Randomized controlled clinical trial
Silvester et al. (2016)	Living gluten-free: Adherence, knowledge, lifestyle adaptation and feelings toward a gluten-free diet	USA	Cross-sectional
Sainsbury et al. (2015)	Dissemination of an online theory-based intervention to improve gluten-free diet adherence in coeliac disease: The relationship between acceptability, effectiveness, and attrition	Australia	Cross-sectional

**Table 3:** Summary of demographics and characteristics of the studies.

Author (date)	Sample size	Female/male	Mean age	Average years since diagnosis	Type of intervention
Akbari Namvar et al. (2022)	130	60%/40%	37.4	4.78 years	Group based education class of 8–10 participants and individualized education program; 3 45 to 60-min sessions focused on celiac disease and a gluten-free diet
Dowd et al. (2022)	41	85%/15%	42	4.32 years	24 individual HIIT exercise sessions plus 6 biweekly education classes aimed at diet and psychoeducation, each for 30–40 min
Martínez-Rodríguez et al. (2021)	28	100%/0%	44.7	n/a	Individually-prescribed diet for each day of the intervention and individual training with a professional 3–4 times a week
Muhammad et al. (2021)	125	77%/23%	52	n/a	Telephone calls targeting celiac disease knowledge and gluten consumption behavior; the average call lasted 49 min and was conducted quarterly
Wolf et al. (2019)	12	75%/25%	30	7.7 years	2-part nutrition education group intervention grounded in social cognitive theory and social determination theory to increase the frequency of home-cooked meals
Haas et al. (2017)	61	69%/31%	16.4	2–5 years	SMS-based TEACH intervention; 45 unique messages sent 2–3 times a week over the period of 3 months
Silvester et al. (2016)	260	85%/15%	55	8.1 years	Evaluation of GF knowledge
Sainsbury et al. (2015)	189	87%/13%	46.5	4.6 years	Bread n’ Butter interactive online intervention; 6 30-min modules over the course of 6 weeks

**Table 4:** Summary of the main results.

Author (date)	Measures	Summary of the results
Akbari Namvar et al. (2022)	Gastrointestinal Symptom Rating Scale (GSRS) SF-36 questionnaire	3 months postintervention GSRS scores significantly lower in the intervention group Quality of life increased in the intervention group after the intervention No changes were observed in the control group
Dowd et al. (2022)	Coeliac Disease Quality of Life (CDQOL) Celiac Disease Adherence Test (CDAT) Godin Leisure Time Exercise Questionnaire Coeliac Disease Gastrointestinal Symptom Rating Scale Self-regulatory efficacy Pittsburgh Sleep Quality Index 12-item self-compassion scale	Significant improvements in quality of life and gastrointestinal symptoms immediately postintervention and 3 months postintervention No significant improvements for adherence to a gluten-free diet or for sleep quality No changes were observed in the control group
Martínez-Rodríguez et al. (2021)	Quality of life measured by WHOQOL-BREF BMI	Mild improvements in postintervention quality-of-life No changes were observed in the control group
Muhammad et al. (2021)	CDAT GFD knowledge CDQOL	Significant improvement in gluten-free diet knowledge and adherence relative to those in the waitlist control group No significant changes were observed in the CDQOL scores nor in dietary burden
Wolf et al. (2019)	CDQOL CDAT Center for Epidemiologic Studies Depression scale (CES-D) State Trait Anxiety Inventory (STAI)	At 1-month follow-up, significant improvements in CDAT, overall CDQOL, depression and anxiety scores No changes in gastrointestinal symptoms
Haas et al. (2017)	GFD adherence Disease symptomatology Patient activation (PAM) CDQOL Blood tests	Significant improvement in enrollment scores compared with 3-month follow-up scores No changes were observed in the control group No changes were observed in serum markers in either group
Silvester et al. (2016)	Gluten-free diet adherence test Gluten-free diet knowledge scale	n/a
Sainsbury et al. (2015)	GFD adherence GFD knowledge CDQOL Psychological symptoms Coping behavior	Intervention made the participants think about or change their behavior Significant improvement in adherence relative to the waitlist control group

**Table 5:** Quality assessment table.

	Selection bias	Study design	Confounders	Blinding	Data collection methods	Withdrawals and drop-outs
Akbari Namvar et al. (2022)	Strong	Strong	Moderate	Moderate	Strong	Strong
Dowd et al. (2022)	Weak	Strong	Moderate	Moderate	Strong	Strong
Martínez-Rodríguez et al. (2021)	Moderate	Strong	Weak	Moderate	Strong	Strong
Muhammad et al. (2021)	Strong	Strong	Weak	Moderate	Strong	Strong
Wolf et al. (2019)	Weak	Strong	Weak	Weak	Moderate	Moderate
Haas et al. (2017)	Moderate	Strong	Weak	Moderate	Strong	Strong
Silvester et al. (2016)	Weak	Moderate	Weak	Moderate	Strong	Strong
Sainsbury et al. (2015)	Weak	Strong	Weak	Moderate	Moderate	Moderate

Most of the studies were focused on the physiological problems associated with celiac disease, gluten-free diet adherence and the impact of celiac disease on the health-related quality of life and mental state of the participants. The results of those studies suggested that there is a lower well-being status and a higher prevalence of anxiety and depression symptoms in patients with celiac disease. Studies in the review employed either randomized controlled clinical trials or cross-sectional designs and were published between 2015 and 2022 Table 2. There were significant differences with regard to the sample size, i.e., Wolf et al. (2019) included only 12 participants, whereas Silvester et al. (2016) had 260 participants Table 3. Only one of the studies used a power calculation to obtain a representative sample size (Akbari Namvar et al., 2022). The participants were invited to participate in the studies through hospitals, local gastrointestinal physician referrals, local celiac support groups and posters in public centers, social media and news advertisements, emails, and word of mouth. In most of the studies, the researchers utilized a waitlist control group, where the participants in the control group were not given any treatment during the study, but they were on a list to receive treatment after the study without any concrete specification of when that treatment would occur (Sainsbury et al., 2015; Haas et al., 2017; Dowd et al., 2019; Muhammad et al., 2020; Martínez-Rodríguez et al., 2021; Akbari Namvar et al., 2022). Only a study by Haas et al. (2017) focused on the younger population, with a mean age of 16.4 (+/− 2.4), while in other studies, the average age of the participants ranged between 30 and 55 years of age Table 3.

Although the procedures of the interventions differed significantly, they all shared some of the same purposes, i.e., to improve the adherence and compliance to a gluten-free diet in those who were diagnosed with celiac disease and to focus not only on the information regarding the gluten-free diet and celiac disease but also on the increasing of patient action, as results from previous studies suggest that only information gathering is not sufficient to cause any sort of significant change in dietary compliance. The subsequent, as well as the individual, purpose of the interventions was to improve the health-related quality of life, which is very much affected by the restrictions of the diagnosis.

### Results regarding the types of interventions in the included studies

There were differences in the interventions employed in the studies Table 3. The researchers in two studies employed group-based education in an education class with a professional dietitian for a limited number of participants (Wolf et al., 2020; Akbari Namvar et al., 2022). In one of those studies, the researchers focused on dietary adherence and suggested that group-based interventions had a significant effect compared to individual education interventions in clinical settings (Akbari Namvar et al., 2022). In the study by Akbari Namvar et al. (2022), the participants attended 3 60-min sessions. The content was designed to improve knowledge regarding celiac disease and its treatment through a gluten-free diet. It also focused on skills in reading and interpreting commercial product labels. During those 3 sessions, participants were not only given lectures but also encouraged to participate in group discussions and skills training. The participants in the study by Wolf et al. (2020) attended two 4.5-h training sessions led by a dietitian and a professional chef to provide nutrition education and to increase the frequency of preparing home-cooked meals. The education was grounded in cognitive behavioral therapy and social determination theory (Wolf et al., 2020).

Two of the studies focused not only on celiac disease and a gluten-free diet but also on physical exercise as part of an overall healthy lifestyle and to increase their overall quality of life (Dowd et al., 2019; Martínez-Rodríguez et al., 2021). In the study by Dowd et al. (2019), the participants attended 12 weeks of high-intensity interval training (HIIT) with a professional HIIT trainer. The classes were held twice a week for 60 min each. In addition, the participants attended six 30 to 40-min group education sessions based on CBT which were held by a trained interventionist. The classes focused on information regarding celiac disease and gluten-free diets, improving psychosocial coping and self-regulatory skills, and learning how to monitor progress, create goals, etc. (Dowd et al., 2019). The participants in the study by Martínez-Rodríguez et al. (2021) also engaged in group physical training for a period of 12 weeks, but the sessions were scheduled 3 times a week for 60 min each. Contrary to the approaches cited above, Martínez-Rodriguez and his colleagues (2021) did not use any education intervention but provided the participants with an individualized prescription diet which was prescribed by a professional dietitian (Martínez-Rodríguez et al., 2021). The prescribed gluten-free diet contained 5 meals a day for each day in a week for the whole period of 12 weeks and was based on diet recommendations for the Spanish population (Martínez-Rodríguez et al., 2021).

Two of the studies used telephone calls to contact the participants (Haas et al., 2017; Muhammad et al., 2021). In a study by Muhammad et al. (2021), the researchers used a nonstructured individual telephone call intervention focused between a participant and two professionals, a gastroenterologist and a clinical nutrition specialist. Prior to the phone call, the participants were given a study document with information about celiac disease and a gluten-free diet. During the phone call, the participants were encouraged to ask questions. The mean call duration was 49 min (Muhammad et al., 2021). In a study by Haas et al. (2017), the researchers used the TEACH program, which consists of 45 unique SMS-based messages developed by the Stanford study team in cooperation with dieticians specialized in celiac disease. These messages were sent 2–3 times a week in the evening over a period of 3 months. Fifteen out of 45 messages included links to other online sources that contained special recipes, restaurant tips, and websites, another 15 contained reasons why the patients should stay on a strict gluten-free diet, and the last 15 contained quiz questions that the participants should respond to Haas et al. (2017).

For one study, the researchers used an interactive computer-based intervention because another study that was focused on that type of intervention revealed that a computer-based program not only promoted increases in knowledge regarding celiac disease and the gluten-free diet but also impacted the behavioral change status of the participants (Meyer et al., 2004). The creation of the intervention took several months, and experts from the fields of psychology, health, and clinical psychology, as well as celiac disease dietitian specialists and some of the patients with celiac disease, cooperated on the creation of the intervention (Sainsbury and Mullan, 2011; Sainsbury et al., 2013, 2015). The intervention consisted of six 30-min modules which should each be completed in a week. The first module was an introductory module that was focused on an explanation of celiac disease, the gluten-free diet and the advantages of adhering to the diet. The second module was focused on the challenges to adhering to a gluten-free diet. It provided the participants with structured problem-solving training for when they encountered problems related to dietary adherence. The third module was focused on diet-related communication and provided typical gluten-free diet situations to help participants communicate assertively toward family, friends, and all other groups. The fourth module employed knowledge and techniques from CBT (cognitive–behavioral therapy) and introduced the participants to the relationship between their thoughts, feelings, and behaviors. It taught them how to use cognitive restructuring in real situations. The fifth module focused on daily life and how to balance daily life while staying on a diet; it included activities such as pleasant activity scheduling associated with a gluten-free diet. Finally, the last six modules combined all the information and knowledge.

Although all the studies referred to some type of intervention in the abstract, a study by Silvester et al. (2016) was focused mostly on knowledge of and adherence to gluten-free diets and the associated burden as measured by The Work and Social Adjustment Scale and The Gluten Free Diet Impact Scale. These scales measured associated behavioral beliefs such as difficulties eating away from home, travel, psychological and physical symptoms, cost, and worries, etc. (Silvester et al., 2016). The researchers found that living gluten-free was prospectively associated with impairments in social leisure activities, increased attention to food and food preparation and emotions such as anxiety, isolation, and frustration (Silvester et al., 2016). That study even revealed that 80% of the 242 participants avoided eating in restaurants, which in turn had a negative social impact on those participants (Silvester et al., 2016).

### Results of included studies in terms of impact on gluten-free adherence and the health-related quality of life

In all the studies, the results showed improvement after completion of the intervention and even after checking the overall improvement after 3–6 months of follow-up.

In a study by Akbar Namvar et al., gastrointestinal symptoms improved 3 months after the intervention; however, immediately after the intervention, there was no significant change observed (Akbari Namvar et al., 2022). These findings correspond to the results found in a study by Muhammad et al., where the researchers suggest that it takes some time to acquire and practice knowledge until it is embedded (Muhammad et al., 2021). Additionally, 87% of participants considered even small accidental consumption of gluten as important to health after the intervention compared to 47% at baseline (Muhammad et al., 2021). Contrary to the hypothesis, there was no statistically significant change in the quality-of-life score or dietary burden score at the 3- or 6-month follow-up (Muhammad et al., 2021).

The quality-of-life score in two studies was observed immediately postintervention and 3 months postintervention (Martínez-Rodríguez et al., 2021; Akbari Namvar et al., 2022). In another study, the intervention group reported significant improvements in the quality of life and gastrointestinal symptoms immediately postintervention, and these improvements were sustained even after 3 months postintervention, although there was no effect on adherence levels to a gluten-free diet (Dowd et al., 2022). The research also showed that increased quality of life and fewer gastrointestinal problems were positively correlated with higher levels of self-compassion in the intervention group (Dowd et al., 2022). In a study by Wolf et al. (2019), the participants at one month’s follow-up had statistically significantly improved their adherence levels and their overall quality of life. Their depression and anxiety scores improved as measured by the CES-D and STAI, respectively (Wolf et al., 2020).

### Results of included studies in terms of impact on gastrointestinal symptoms

In contrast, there were no changes in gastrointestinal symptoms, probably due to only one month of follow-up testing, which was not enough time for the intestine to heal (Wolf et al., 2020). Additionally, most of the participants asked for additional classes, and all agreed that the intervention was both practical and very helpful (Wolf et al., 2020). In a study by Haas et al. (2017), the researchers found that patient engagement and self-management improved in those who received the treatment and that the intervention helped them with issues such as feelings of social isolation and fears of misunderstanding celiac disease. However, no change in serum markers in TTG IgA and DGP IgA associated with celiac disease was identified (Haas et al., 2017). The authors suggested that such a short time was probably not sufficient for a blood test to show any significant results, and they recommended using this platform for a further longitudinal study design that would provide a better understanding of the success of such a program (Haas et al., 2017). That study was the only one that also employed objective measurements *via* blood tests (Haas et al., 2017); all of the other studies used self-report questionnaires (Wolf et al., 2020; Muhammad et al., 2021; Akbari Namvar et al., 2022; Dowd et al., 2022) or self-report questionnaires and professional nutrition specialists (Sainsbury et al., 2015; Martínez-Rodríguez et al., 2021). In the study by Sainsbury and colleagues, the intervention resulted in improvements in dietary adherence, and it also helped the participants to better understand celiac disease and the gluten-free diet (Sainsbury et al., 2013, 2015), and all participants reported that their physical and psychological quality of life increased. Notably, the completion rate reached only 50% (as the drop-out rates were very high); therefore, the generalization of those results should be made with caution (Sainsbury et al., 2013, 2015).

Overall, the results of these studies showed statistically significant benefits of any intervention for celiac disease on gluten-free dietary adherence, although the administration and procedures of the interventions differed.

## Discussion

One of the main aims of the present systematic review was to get a perspective about interventions for patients with coeliac disease to increase the adherence to a gluten-free diet and to increase the health-related quality of life. We have extensively searched four databases, i.e., Scopus, Web of Science, PubMed and ProQuest, which resulted in the inclusion of eight studies that introduced interventions for this purpose. We can state that the number of possible interventions designed for adults with celiac disease that need to adhere to a life-long gluten-free diet is quite limited compared to the number of studies that focus on psychological and physiological problems associated with celiac disease. Most of these studies are cross-sectional studies that focus on the adverse effects of gluten-free diets and celiac disease on diet adherence, quality of life and higher incidences of depression and anxiety, predominantly performed by medical doctors in the research teams (Ciclitira et al., 2005; Woodward, 2011; Ford et al., 2012; Green et al., 2015).

The intervention designs differ among the selected studies, although three types of interventions, i.e., group-based, telephone-based, and online-based interventions, form the basis of interventions for patients with celiac disease. In some studies, the researchers used a combined form, where, for example, one part of the intervention was delivered online and the other part was delivered in-person. The studies share a focus on education, where the main purpose of the educational part was to increase the general awareness of celiac disease as well as to provide an explanation of reasons to adhere to a gluten-free diet. Some of the studies also focused on the improvement of physical health and implemented regular exercise (Martínez-Rodríguez et al., 2021; Dowd et al., 2022) in addition to providing tools for preparing gluten-free meals at home (Wolf et al., 2020). We can see that a multidisciplinary intervention approach might be beneficial.

Regarding the effects of those interventions on the gluten-free adherence rate, all the clinical controlled trial studies showed significant improvements in the experimental group post-intervention as well as 3 months after intervention, whereas no change was found in the waitlist-control group. Moreover, the knowledge about celiac disease and the gluten-free diet increased after the intervention. Considering the only effective treatment for celiac disease is following a gluten-free diet (Ciclitira et al., 2005; Ford et al., 2012; Mehta et al., 2018) and that strict adherence to a gluten-free diet is positively correlated to a better perceived health-related quality of life (Casellas et al., 2008; 2015), the patients might benefit from participating in various interventions. It is not surprising that the results of the studies in the systematic review indicated a positive effect of interventions on the health-related quality of life in most of the studies; however, in one study (Muhammad et al., 2021), the experimental group of participants scored lower on their satisfaction with life in dietary burdens and worries and concerns at 3 months post-intervention. The studies that showed improvement in both the gluten-free adherence scores and health-related quality of life employed a combination of methods, whereas the study intervention by Muhammad et al. (2021) consisted of 2 unstructured telephone calls with dietitians. Therefore, the direction of future research in the area of intervention for patients with celiac disease might benefit from using more structured methods for sharing knowledge in terms of information about celiac disease, diet adherence and the benefits of that adherence.

It seems that despite the inconsistencies in the procedures of various intervention programs, it could be stated that any intervention might be beneficial for those with celiac disease and a need to adhere to a lifelong gluten-free diet. The results of those studies, however, suggest that the combination of the platforms, i.e., online and/or telephone platform with a group-focused in-person education component might bring more satisfactory outcomes. Moreover, the potential benefits of the interventions conducted by a multi-disciplinary team represented by gastroenterologists, psychologists, dietitians, and other professionals might bring more positive outcomes for patients with celiac disease.

## Conclusion

The implications for the practice of interventions for patients with celiac disease is significant. Overall, an intervention designed for chronically ill adults with celiac disease supports their adherence to a gluten-free diet, improves their quality of life, helps them overcome anxieties associated with travelling, eating outside the home, and shopping for food, and helps them decrease their level of stress and anxiety associated with the diet. Future studies should probably combine online and/or telephone platform interventions with group-based interventions to promote behavioral changes among patients with celiac disease and other chronic types of disease and could potentially bring about change in the current health system.

Regarding the possible interventions, it seems that the combination of methods might be a good option for adults with celiac disease who represent a diverse group with specific needs and preferences. Those changes might also eventually lead to changes in health insurance company policies to start supporting those that need to adhere to a gluten-free diet, as almost 51.3% of participants stated that associated costs are the burden of the disease, often resulting in non-compliance to the diet. Although, the current situation in the Czech Republic regarding supporting patients with celiac disease is very limited and the contributions are low, if any. Furthermore, there is no research in this area showing benefits of implementing those interventions in the healthcare system.

As the results of this systematic review suggest, the targeted intervention should combine the knowledge from various disciplines and explain the condition, provide dietitian information about the diet, and include some cognitive behavioral therapy techniques that would help individuals stay gluten-free.

## Author contributions

MP performed the literature search and the preparation and final editing of the manuscript. MS supervised the literature search and the preparation of the final version. PŘ performed the literature search. MA performed the final editing of the manuscript and secured the funding for the research. RP performed the final editing. All authors contributed to the article and approved the submitted version.

## Funding

This work was supported by the Cooperatio Program, research area: Psychology.

## Conflict of interest

The authors declare that the research was conducted in the absence of any commercial or financial relationships that could be construed as a potential conflict of interest.

## Publisher’s note

All claims expressed in this article are solely those of the authors and do not necessarily represent those of their affiliated organizations, or those of the publisher, the editors and the reviewers. Any product that may be evaluated in this article, or claim that may be made by its manufacturer, is not guaranteed or endorsed by the publisher.
