# Supplementary material for: The effectiveness of gluten-free dietary interventions: A systematic review
Source: Front Psychol. 2023 Mar 22;14:1107022. doi: 10.3389/fpsyg.2023.1107022 (PMC10075251; doi:10.3389/fpsyg.2023.1107022)
Supplement: Supplementary file 1 [file Table_1.DOCX]

Supplementary material:

*Detailed Component Ranking – Quality Assessment Tool for Quantitative Studies*

| Question | | Author (date) | | | | | | | |
| --- | --- | --- | --- | --- | --- | --- | --- | --- | --- |
|  | Akbari Namvar et al. (2022) | | Dowd et al. (2021) | Martínez-Rodríguez et al. (2021) | Muhammad et al. (2021) | Wolf et al. (2019) | Haas et al. (2017) | Silverster et al. (2016) | Sainsbury et al. (2015) |
| 1. Selection Bias | | | | | | | | | |
| *Q1. Are the individuals selected to participate in the study likely to be representative of the target populations?* |  | |  |  |  |  |  |  |  |
| 1. Very likely | x | |  |  |  |  |  |  |  |
| 2. Somewhat likely |  | |  |  | x |  | x | x |  |
| 3. Not likely |  | | x | x |  |  |  |  | x |
| 4. Can’ tell |  | |  |  |  | x |  |  |  |
| *Q2. What percentage of selected individuals agreed to participate?* |  | |  |  |  |  |  |  |  |
| 1. 80-100% | x | |  | x | x |  |  |  |  |
| 2. 60-79% |  | |  |  |  |  | x |  |  |
| 3. Less than 60% |  | |  |  |  |  |  |  | x |
| 4. Not applicable |  | |  |  |  |  |  |  |  |
| 5. Can’t tell |  | | x |  |  | x |  | x |  |
| Rate this section | Strong | | Weak | Moderate | Strong | Weak | Moderate | Weak | Weak |
| 1. Study design | | | | | | | | | |
| *Indicate the study design* |  | |  |  |  |  |  |  |  |
| 1. Randomized controlled trial | x | | x | x | x |  | x |  | x |
| 2. Controlled clinical trial |  | |  |  |  | x |  |  |  |
| 3. Cohort analytic |  | |  |  |  |  |  |  |  |
| 4. Case-control |  | |  |  |  |  |  |  |  |
| 5. Cohort |  | |  |  |  |  |  |  |  |
| 6. Interrupted time series |  | |  |  |  |  |  |  |  |
| 7. Other specify |  | |  |  |  |  |  |  |  |
| 8. Can’t tell |  | |  |  |  |  |  | x |  |
| *Was the study described as randomized? If NO, go to Component C.* |  | |  |  |  |  |  |  |  |
| No |  | |  |  |  | x |  | x | x |
| Yes | x | | x | x | x |  | x |  |  |
| *If Yes, was the method of randomization described?* |  | |  |  |  |  |  |  |  |
| No |  | | x |  | x |  |  |  |  |
| Yes | x | |  | x |  |  | x |  |  |
| *If Yes, was the method appropriate?* |  | |  |  |  |  |  |  |  |
| No |  | |  |  |  |  |  |  |  |
| Yes | x | |  | x |  |  | x |  |  |
| Rate this section | Strong | | Strong | Strong | Strong | Strong | Strong | Moderate | Strong |
| 1. Confounders | | | | | | | | | |
| *Q1. Were there important differences between groups prior to the intervention?* |  | |  |  |  |  |  |  |  |
| 1. Yes |  | | x |  |  |  |  |  |  |
| 2. No | x | |  | x |  |  |  |  |  |
| 3. Can’t tell |  | |  |  | x | x | x | x | x |
| *Q2. If yes, indicate the percentage of relevant confounders that were controlled (either in the design (e.g. stratification, matching) or analysis?* |  | |  |  |  |  |  |  |  |
| 1. 80-100% (most) |  | |  |  |  |  |  |  |  |
| 2. 60-79% (some) |  | |  |  |  |  |  |  |  |
| 3. Less than 60% (few or none) |  | |  |  |  |  |  |  |  |
| 4. Can’t tell |  | | x | x | x |  | x |  |  |
| Rate this section | Moderate | | Moderate | Weak | Weak | Weak | Weak | Weak | Weak |
| 1. Blinding | | | | | | | | | |
| *Q1. Was (were) the outcome assessor(s) aware of the intervention or exposure status of participants?* |  | |  |  |  |  |  |  |  |
| 1. Yes |  | | x | x | x |  | x | x | x |
| 2. No | x | |  |  |  |  |  |  |  |
| 3. Can’t tell |  | |  |  |  | x |  |  |  |
| *Q2. Were the study participants aware of the research question?* |  | |  |  |  |  |  |  |  |
| 1. Yes |  | |  |  |  |  |  |  |  |
| 2. No |  | |  |  |  |  |  |  |  |
| 3. Can’t tell | x | | x | x | x | x | x | x | x |
| Rate this section | Moderate | | Moderate | Moderate | Moderate | Weak | Moderate | Moderate | Moderate |
| 1. Data collection methods | | | | | | | | | |
| *Q1. Were data collection tools shown to be valid?* |  | |  |  |  |  |  |  |  |
| 1. Yes | x | | x | x | x |  | x | x | x |
| 2. No |  | |  |  |  | x |  |  |  |
| 3. Can’t tell |  | |  |  |  |  |  |  |  |
| *Q2. Were data collection tools shown to be reliable?* |  | |  |  |  |  |  |  |  |
| 1. Yes |  | | x |  |  |  |  |  |  |
| 2. No |  | |  |  |  |  |  |  |  |
| 3. Can’t tell | x | |  | x | x | x | x | x | x |
| Rate this section | Strong | | Strong | Strong | Strong | Moderate | Strong | Strong | Moderate |
| 1. Withdrawals and drop-outs | | | | | | | | | |
| *Q1. Were withdrawals and drop-outs reported in terms of numbers and/or reasons per group?* |  | |  |  |  |  |  |  |  |
| 1. Yes | x | | x | x | x |  | x | x | x |
| 2. No |  | |  |  |  | x |  |  |  |
| 3. Can’t tell |  | |  |  |  |  |  |  |  |
| 4. Not Applicable |  | |  |  |  |  |  |  |  |
| *Q2. Indicate the percentage of participants completing the study* |  | |  |  |  |  |  |  |  |
| 1. 80-100% | x | | x | x | x |  | x | x |  |
| 2. 60-79% |  | |  |  |  |  |  |  |  |
| 3. Less than 60% |  | |  |  |  |  |  |  | x |
| 4. Can’t tell |  | |  |  |  | x |  |  |  |
| 5. Not Applicable |  | |  |  |  |  |  |  |  |
| Rate this section | Strong | | Strong | Strong | Strong | Moderate | Strong | Strong | Moderate |
| 1. Intervention Integrity | | | | | | | | | |
| Q1. What percentage of participants received the allocated intervention or exposure of interest? |  | |  |  |  |  |  |  |  |
| 1. 80-100% | x | | x | x | x | x | x | x |  |
| 2. 60-79% |  | |  |  |  |  |  |  |  |
| 3. Less than 60% |  | |  |  |  |  |  |  |  |
| 4. Can’t tell |  | |  |  |  |  |  |  | x |
| *Q2. Was the consistency of the intervention measured?* |  | |  |  |  |  |  |  |  |
| 1. Yes |  | |  |  |  |  |  |  |  |
| 2. No |  | |  |  |  |  |  |  |  |
| 3. Can’t tell | x | | x | x | x | x | x | x | x |
| *Q3. Is it likely that subjects received an unintended intervention that my influence the results?* |  | |  |  |  |  |  |  |  |
| 1. Yes |  | |  |  |  |  |  |  |  |
| 2. No |  | |  |  |  |  |  |  |  |
| 3. Can’t tell | x | | x | x | x | x | x | x | x |
| 1. Analyses | | | | | | | | | |
| *Q1. Indicate the unit of allocation* |  | |  |  |  |  |  |  |  |
| Community |  | |  |  |  | x |  |  |  |
| Organization/institution | x | | x | x | x |  | x | x | x |
| Practice/office |  | |  |  |  |  |  |  |  |
| Individual |  | |  |  |  |  |  |  |  |
| *Q2. Indicate the unit of analysis* |  | |  |  |  | x |  |  |  |
| Community | x | | x | x | x |  | x | x | x |
| Organization/institution |  | |  |  |  |  |  |  |  |
| Practice/office |  | |  |  |  |  |  |  |  |
| Individual |  | |  |  |  |  |  |  |  |
| *Q3. Are the statistical methods appropriate for the study design?* |  | |  |  |  |  |  |  |  |
| 1. Yes | x | | x | x | x | x | x | x | x |
| 2. No |  | |  |  |  |  |  |  |  |
| 3. Can’t tell |  | |  |  |  |  |  |  |  |
| *Q3. Is the analysis performed by intervention allocation status (i.e. intention to treat) rather than the actual intervention received?* |  | |  |  |  |  |  |  |  |
| 1. Yes | x | | x | x | x | x | x | x | x |
| 2. No |  | |  |  |  |  |  |  |  |
| 3. Can’t tell |  | |  |  |  |  |  |  |  |
